# Supplementary material for: Association between Altered Blood Parameters and Gut Microbiota after Synbiotic Intake in Healthy, Elderly Korean Women
Source: Nutrients. 2020 Oct 12;12(10):3112. doi: 10.3390/nu12103112 (PMC7650560; doi:10.3390/nu12103112)
Supplement: Supplementary file 1 [file nutrients-12-03112-s001.zip › Table S7.pdf]

Table S7. Relative abundance and *P-value* of intestinal microbial phyla (A), genera (B), and species (C) in 37 participants based on 16S rRNA analysis.

	Baseline	After	Washout	*P*-value	
				Baseline vs After	After vs Washout
**Phylum level**					
Proteobacteria	1.2%	1.1%	2.8%	0.077	0.341
Actinobacteria	7.77%	9.01%	5.85%	0.118	0.024
Bacteroidetes	5.30%	8.47%	5.93%	0.026	0.03
Firmicutes	85.5%	81.3%	84.9%	0.074	0.108
ETC (under 1% in average)	0.23%	0.14%	0.47%		
**Genus level**					
Parabacteroides		1.03%		5.60E-05	7.40E-05
Prevotella		1.10%		0.871	0.854
Streptococcus	1.74%	1.11%	1.21%	0.63	0.987
Dorea	2.04%	1.36%	2.00%	0.089	0.137
Subdoligranulum	1.62%	1.90%	1.69%	0.331	0.168
Agathobacter	2.33%	1.91%	2.34%	0.725	0.662
Eubacterium_g5	3.30%	2.93%	3.90%	0.189	0.008
Fusicatenibacter	4.06%	5.10%	3.72%	0.039	0.014
Anaerostipes	5.86%	5.43%	7.32%	0.6	0.089
Bacteroides	3.62%	5.55%	4.37%	0.143	0.155
Bifidobacterium	6.92%	8.36%	5.30%	0.099	0.017
Faecalibacterium	9.25%	13.76%	11.07%	0.009	0.165
Blautia	23.69%	19.28%	25.15%	0.116	0.005
Lachnospiraceae_uc	1.06%		1.02%	0.003	0.002
Lactobacillus	2.01%		1.68%	0.825	0.725
Ruminococcus_g2	1.64%	1.36%	1.69%	0.57	0.534
Ruminococcus_g4	1.53%	1.09%	1.54%	0.019	0.036
**Species level**					
B. angulatum		0.01%		0.196	0.195
B. animalis		0.18%		4.20E-13	2.00E-12

B. longum	0.47%	1.16%	0.44%	0.14	0.035
B. pseudolongum		0.01%		2.50E-08	6.50E-08
L. paracasei		0.01%		1.80E-07	2.00E-07
L. plantarum		0.01%		2.40E-04	2.30E-05
