# Supplementary material for: Association between Altered Blood Parameters and Gut Microbiota after Synbiotic Intake in Healthy, Elderly Korean Women
Source: Nutrients. 2020 Oct 12;12(10):3112. doi: 10.3390/nu12103112 (PMC7650560; doi:10.3390/nu12103112)
Supplement: Supplementary file 1 [file nutrients-12-03112-s001.zip › Table S4.pdf]

Table S4. Comparison of biochemical markers of safety (mean ± SD) at baseline and at the endpoint of the trial.

Safety parameter indices		Baseline	After	Washout	Baseline vs After P-value	After vs Washout P-value
Hematologic	WBC	5.81 ± 1.51	5.59 ± 1.04	5.43 ± 1.12	0.512	0.202
	RBC	4.24 ± 0.49	4.16 ± 0.50	4.14 ± 0.48	0.180	0.564
	HGB	13.16 ± 1.07	13.11 ± 1.15	12.92 ± 1.06	0.617	0.071
	HCT	39.16 ± 2.74	38.65 ± 3.03	38.08 ± 2.89	0.280	0.061
	MCV	90.97 ± 3.22	91.05 ± 3.19	90.92 ± 3.17	0.795	0.310
	MCH	30.73 ± 1.43	30.95 ± 1.33	31.00 ± 1.41	0.046	0.527
	MCHC	33.73 ± 0.99	33.89 ± 0.77	34.19 ± 0.97	0.083	0.012
	PLT	229.24 ± 46.86	229.11 ± 44.61	220.59 ± 50.15	0.952	0.058
	RDW-SD	42.19 ± 2.22	42.24 ± 2.50	12.54 ± 0.65	0.855	0.002
	RDW-CV	12.68 ± 0.63	12.73 ± 0.69	41.54 ± 2.27	0.414	0.008
	PDW	12.30 ± 1.90	11.92 ± 1.67	11.81 ± 1.71	0.063	0.559
	MPV	10.43 ± 0.99	10.27 ± 0.84	10.46 ± 0.96	0.134	0.052
	P-LCR	29.03 ± 7.95	27.08 ± 6.53	27.95 ± 7.04	0.003	0.082
Kidney	BUN	16.30 ± 4.19	15.65 ± 3.81	14.78 ± 4.10	0.261	0.153
	UA	4.57 ± 1.12	4.43 ± 1.14	4.30 ± 1.10	0.282	0.275
	TP	7.73 ± 0.61	7.43 ± 0.55	7.59 ± 0.64	0.001	0.034
Urine test	BLD	0.66 ± 1.60	0.51 ± 1.28	0.59 ± 1.36	0.461	0.546
	BIL	0.68 ± 1.31	0.24 ± 0.83	0.30 ± 1.02	0.083	0.593
	URO	1.11 ± 0.46	1.08 ± 0.49	1.05 ± 0.33	1.000	0.317
	KET	1.14 ± 1.32	1.00 ± 1.13	1.11 ± 1.22	0.774	0.573
	pH	6.45 ± 0.56	6.27 ± 0.43	6.38 ± 0.53	0.102	0.246
	S. G	1.02 ± 0.01	1.02 ± 0.00	1.02 ± 0.01	0.089	0.174
Liver	LFU	1.73 ± 1.98	1.65 ± 2.02	1.84 ± 2.11	0.734	0.483
	AST	26.14 ± 5.15	26.03 ± 5.71	26.41 ± 6.84	0.898	0.864
	ALT	21.30 ± 8.43	22.73 ± 10.10	22.27 ± 9.62	0.396	0.670
	T-BIL	0.89 ± 0.46	0.86 ± 0.42	0.81 ± 0.57	0.705	0.480
	GGT	22.35 ± 10.35	26.95 ± 19.35	23.46 ± 11.99	0.015	0.009
	ALB	4.00 ± 0	4.03 ± 0.16	4.03 ± 0.16	0.317	1.000
	A/G ratio	1.05 ± 0.23	1.43 ± 0.50	1.14 ± 0.35	0.000	0.002
	B/C ratio	28.00 ± 6.90	28.14 ± 6.07	26.76 ± 7.06	0.887	0.213

Abbreviations: WBC, white blood cell; RBC, red blood cell; HGB, hemoglobin; HCT, hematocrit; MCV, mean corpuscular volume; MCH, mean corpuscular hemoglobin; MCHC, mean corpuscular hemoglobin concentration; PLT, platelet; RDW-SD, red cell distribution width standard deviation; RDW-CV, red cell distribution width coefficient of variation; PDW, platelet distribution width; MPV, mean platelet volume; P-LCR, platelet-large cell ratio; BUN, blood urea nitrogen; UA, uric acid; TP, total protein; BLD, blood; BIL, bilirubin; URO, urobilinogen; KET, ketone body; pH, potential of hydrogen; S.G, specific gravity; LFU, leukocyte forming unit; AST, aspartate aminotransferase; ALT, alanine aminotransferase; T-BIL, total-bilirubin; GGT, gamma-glutamyl transpeptidase; ALB, albumin; A/G ratio, albumin/globulin ratio; B/G ratio, BUN/creatinine ratio.

Urine test positive and negative values were converted to numbers (Urine test: 0, negative; 1, normal; 2, trace; 3, +; 4, ++; 5, +++).

P-values are based on the paired t-test and Wilcoxon rank-sum test.
